# Attitudes and Perceptions of Dentists and Dental Residents Practicing in the Navi Mumbai Region Toward the Use of Artificial Intelligence in Dentistry: A Descriptive Survey

**DOI:** 10.7759/cureus.66836

**Published:** 2024-08-14

**Authors:** Aarti S Bedia, Sayem A Mulla, Amit Patil, Sumit V Bedia, Mahesh Ghadage, Sheetal Mali

**Affiliations:** 1 Oral Medicine and Radiology, Bharati Vidyapeeth (Deemed to be University) Dental College and Hospital, Navi Mumbai, IND; 2 Dentistry, Bharati Vidyapeeth (Deemed to be University) Dental College and Hospital, Navi Mumbai, IND; 3 Conservative Dentistry and Endodontics, Bharati Vidyapeeth (Deemed to be University) Dental College and Hospital, Navi Mumbai, IND; 4 Prosthodontics, Bharati Vidyapeeth (Deemed to be University) Dental College and Hospital, Navi Mumbai, IND

**Keywords:** clinical dentistry, dental clinics, ai in dentistry, machine learning, dental disease diagnosis, artificial intelligence

## Abstract

Introduction

Artificial intelligence (AI) has been gaining considerable attention in recent years within the healthcare field. It has established a presence in various aspects of health sciences, including accurate diagnosis and precise, streamlined treatment. This study aimed to assess the attitudes of dental residents and dentists in the Navi Mumbai region toward the use of AI in dentistry.

Methods

An online questionnaire-based survey was conducted, inviting 130 dental residents and dentists from the Navi Mumbai region. The collected data were compiled on a worksheet and subjected to descriptive statistical tests, which were expressed in numbers and frequencies.

Results

A total of 100 responses were received. Sixty-eight percent of individuals agreed that AI helps enhance diagnosis and treatment planning in the dental field. Sixty-five percent of the respondents stated that they are most likely to incorporate AI tools into their practice within the next five years.

Conclusion

From the present study, it can be inferred that AI is a promising and essential subsidiary tool in dentistry as well as in healthcare as a whole. However, major concerns such as extensive, in-depth training, data security, and cybercrime must be addressed before the full-scale incorporation of AI in the health sciences.

## Introduction

The concept of artificial intelligence (AI) was conceived in 1943, but the term was coined by John McCarthy at a conference in 1956. The concept revolved around creating machines that could replicate human tasks [1,2]. AI is a software system that uses data sources to make independent decisions or assist humans in decision-making. It encompasses machine learning, representation learning, deep learning, and natural language processing. AI is a branch of computer science that analyzes massive amounts of data. However, it is not only related to computer science; it also finds applications in various health science specialties such as medicine, dentistry, philosophy, psychology, linguistics, and statistics [3,4]. In the context of the health sciences, particularly dentistry, researchers and dentists can use AI algorithms to identify novel therapeutic targets, predict outcomes and prognoses, optimize treatment techniques, and enhance patient care and dental professionalism [5].

Recently, the use of AI in various healthcare sectors has significantly increased. AI is extremely useful in continuously assessing and monitoring a patient's health, understanding the long-term effects of drugs, and anticipating health-related risks. AI has the potential to drastically reduce the long hours spent by dental professionals. Additionally, it is possible to enhance people's health at a lower cost, provide personalized, preventive, and predictive dentistry, and integrate healthcare for all. Most importantly, AI has the potential to improve dental care standards, enhance diagnostic accuracy and effectiveness, create better visuals for treatment, simulate results, and predict oral diseases and health outcomes [6,7]. AI models have also gained popularity as auxiliary tools for improving diagnostic precision and accuracy. AI technology has been widely applied in the field of medical sciences, where it has demonstrated excellent performance in various patient-care tasks, such as disease diagnosis and assessing a patient's risk of developing a disease, among many others [8-10].

Just as every coin has two sides, the use of AI in the healthcare sector comes with both advantages and disadvantages. AI-powered systems raise significant concerns about data security and privacy. Health records are particularly important and vulnerable, making them frequent targets for hackers during data breaches. The lack of standardized guidelines for the ethical use of AI in healthcare has further exacerbated the situation. Data privacy, social concerns, ethical issues, hacking threats, and challenges faced by developers are among the obstacles to successfully implementing AI in the medical sector. Therefore, it is critical to address these barriers before fully integrating AI into healthcare [11]. This article aims to understand the perception and attitude of dentists and dental residents practicing routine dental procedures regarding the implementation of AI in dentistry.

## Materials and methods

Study design and setting

The present study was a cross-sectional survey conducted among dentists and dental residents practicing routine dentistry (both clinical and diagnostic) in the Navi Mumbai region. Informed consent was obtained from each participant.

Inclusion criteria and exclusion criteria

Dentists and dental residents who are actively practicing routine dental procedures or are involved in routine dental processes are included in the study. Dental students were excluded from the present study because they have not yet begun actively practicing routine dentistry.

Questionnaire design

The questionnaire consisted of a first section that collected informed consent and demographic details, such as gender and qualification. It then proceeded to gather responses, gauging attitudes and perceptions toward the use of AI in dentistry (Table 1). The questionnaire was validated by six subject experts and subsequently piloted, yielding a Content Validity Index score of 0.80 for all questions. It was distributed electronically to collect a target of 130 responses based on a convenient sampling method.

**Table 1:** Questionnaire form used AI: artificial intelligence, CBCT: cone beam computed tomography, MRI: magnetic resonance imaging

Survey title: Attitudes and perceptions of dentists and dental residents practicing in the Navi Mumbai region toward the use of AI in dentistry: a descriptive survey		
Question number	Question	Variables/options
1.	Do you consent to participate in this study?	Yes
		No
2.	Qualification?	Intern
		Postgraduate resident
		Dentist (clinical)
		Dentist (non-clinical)
		Others (please specify)
3.	How familiar are you with AI?	Very familiar
		Somewhat familiar
		Not very familiar
		Not at all familiar
4.	What types of AI technology have you used or been involved in the development of (select all that apply)?	Diagnostic software
		Treatment planning software
		Imaging and radiology software
		Patient monitoring software
		Others (please specify)
5.	How effective do you believe AI technology is in assisting with dental diagnosis and treatment planning?	Very familiar
		Somewhat familiar
		Not very familiar
		Not at all familiar
6.	In what ways do you believe AI technology could improve dental practice (select all that apply)?	Improved accuracy of diagnosis
		Improved treatment planning
		Streamlining of administrative tasks
		Improved patient outcomes
		Others (please specify)
7.	How concerned are you about the impact of AI technology on job security in the dental field?	Very concerned
		Somewhat concerned
		Not very concerned
		Not at all concerned
8.	How likely are you to incorporate AI technology into your dental practice in the next 5 years?	Very likely
		Somewhat likely
		Not very likely
		Not at all likely
9.	What factors do you believe will influence the adoption of AI technology in the dental field (select all that apply)?	Cost of technology
		Ease of use
		Availability of training and support
		Regulatory and legal considerations
		Patient acceptance
		Others (please specify)
10.	AI can be used in oral radiology for the interpretation of various radiographs (CBCT, MRI, etc.) for the differentiation between vital and pathological signs. Do you agree?	Yes
		No
		Maybe
		Others (please specify)
11.	AI can be used in oral pathology and microbiology for the interpretation of different tissue specimens including neoplasm. Do you agree?	Yes
		No
		Maybe
		Others (please specify)
12.	In your opinion, are there any ethical concerns associated with the use of AI technology in dentistry?	Please specify

Statistical analysis

The data collected was compiled on a worksheet and expressed as numbers and percentages. Descriptive statistics were used for data interpretation.

## Results

A total of 100 responses were collected from this online survey. Of these, 52 were dental residents, and the remaining 48 were dentists. Among the dentists, 18 were from non-clinical branches involved in the diagnostic aspect of dentistry, while the remaining 30 were engaged in the clinical aspect of dentistry.

**Figure 1:**
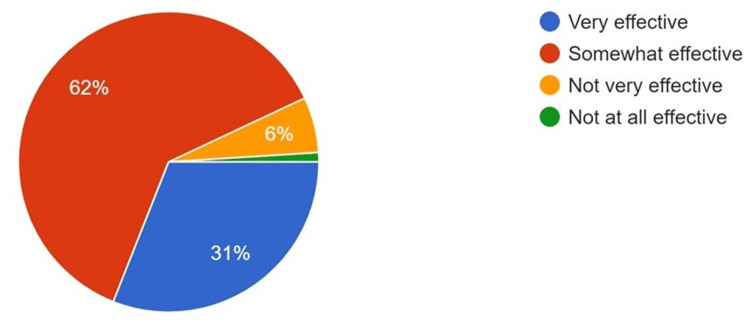
Responses to the effectiveness of AI in dental diagnosis and treatment planning AI: artificial intelligence

A total of 56% of the respondents stated that they were familiar with AI, while 25% were not very familiar with it. Sixty-five percent of the personnel had used imaging and radiology software as an AI tool, followed by treatment planning software (45%) and patient monitoring software (35%). The majority of respondents (68% and 67%, respectively) believed that AI could improve treatment planning and enhance the accuracy of diagnosis (Figure 1). When asked about the factors that would influence their decision to incorporate AI into routine dental practice, 74% stated that the cost of technology would be the most crucial factor, followed by ease of use and availability of training and support (69% each), regulatory and legal considerations (50%), and patient acceptance (49%).

**Figure 2:**
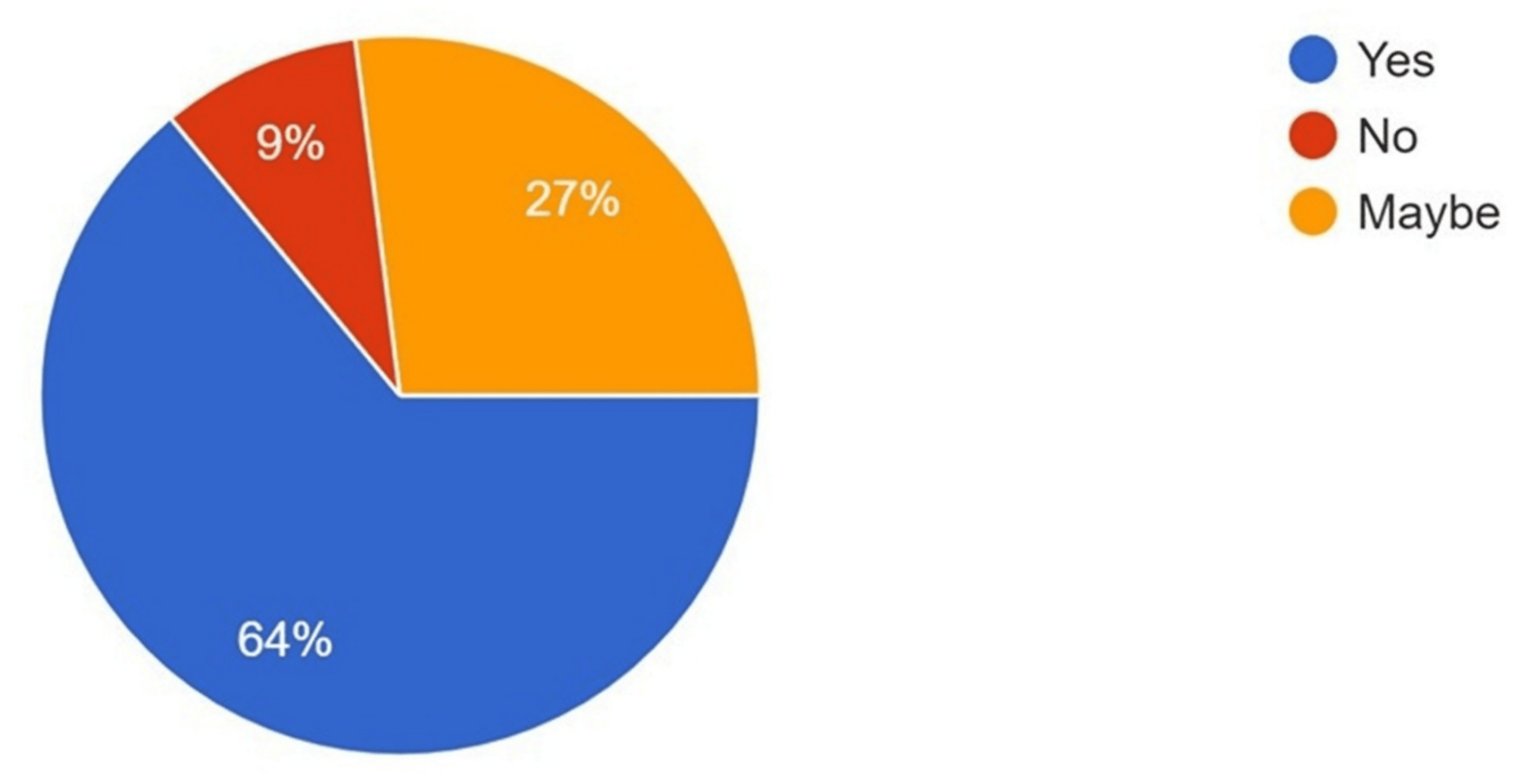
Responses to the use of AI in oral pathology and microbiology AI: artificial intelligence

**Figure 3:**
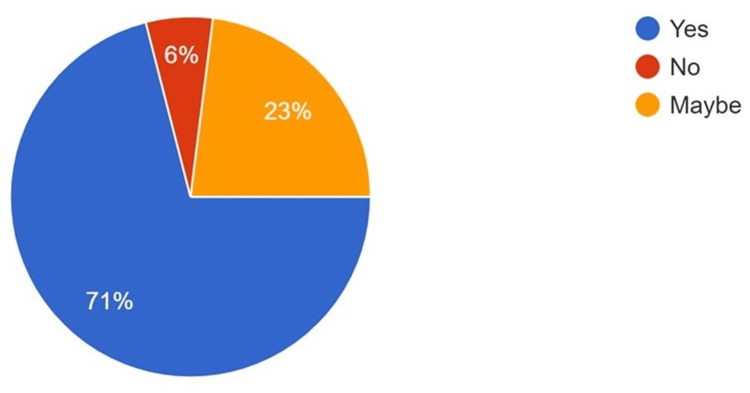
Responses to the use of AI in oral radiology AI: artificial intelligence

**Figure 4:**
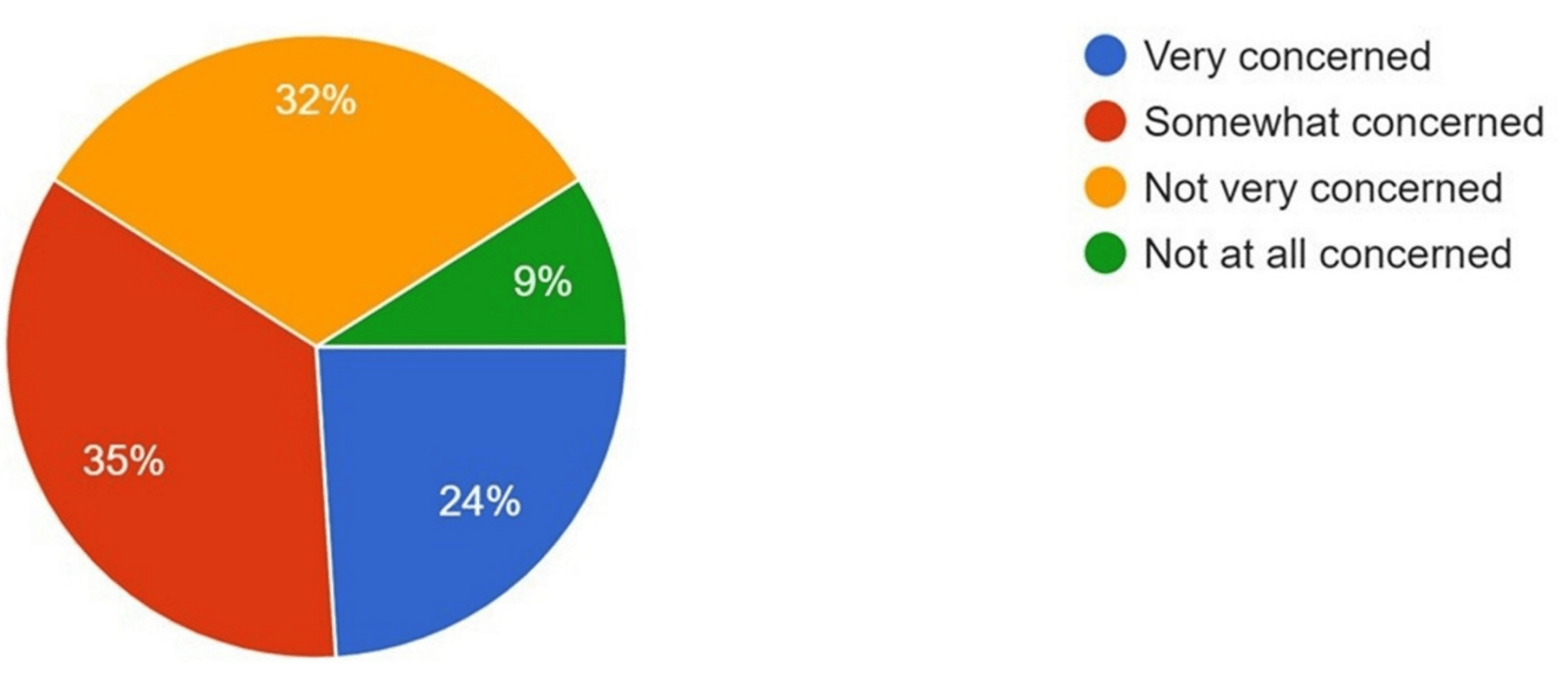
Concerns of respondents with regard to job security in implementing AI in the dental field AI: artificial intelligence

**Figure 5:**
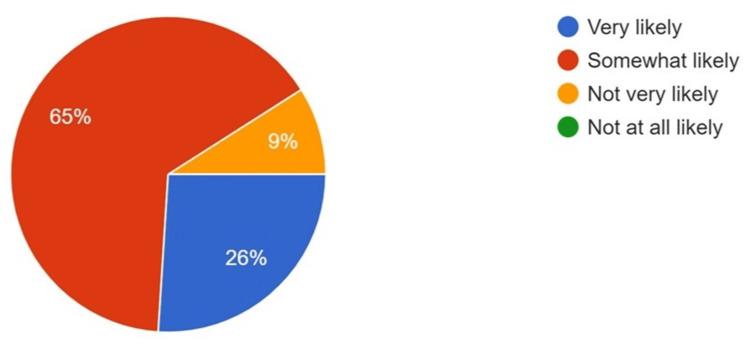
Responses to the incorporation of AI in the dental field in the next five years AI: artificial intelligence

The majority of the participants believed that AI technology aids in the interpretation of various head, face, and neck pathologies when used in conjunction with oral pathology (64%) and oral radiology (71%) (Figures 2-3). Conversely, when asked about the impact of AI on job security in the dental field, a mixed response was obtained, although most participants had some concern regarding AI and job security (Figure 4). However, 65% of respondents stated that they are most likely to incorporate AI into their routine, day-to-day practice within the next five years (Figure 5).

The last section of the present survey included an open-ended question asking for any particular comments regarding the use of AI in dentistry or healthcare at large. Various comments were received, stating that AI is a promising tool that can assist healthcare professionals if used wisely, although total reliance on it can never be a complete solution. Other comments addressed concerns such as monitoring AI, as it is prone to hacking, and limitations such as data accuracy.

## Discussion

In terms of complexity, diversity, and computational capabilities, AI has advanced rapidly, particularly in the health sciences [12]. It has emerged as one of the most promising healthcare technologies, with significant progress in predictive machine-learning models for dental care [13]. AI has the potential to be applied to dental practices and play a significant role in practice management. Dentists may use AI systems as a supplement to provide precise dental diagnoses and treatment planning, as well as for early detection of dental conditions, resulting in improved patient outcomes [14]. In the present survey, more than half of the respondents (56%) reported being familiar with the applicability of AI in the dental field. However, it should be noted that 44% of respondents were either not familiar with or only slightly familiar with its applicability in dentistry. These results contrast with a study conducted by Roganović et al., where 112 (58.3%) participants were partly familiar with the use of AI in healthcare, while only 21.7% were totally unfamiliar and 14.6% were familiar with it [15]. A similar contrast was found in a study by Eschert et al. [16].

With the advent of AI, one major concern that has arisen is whether it could replace or negatively impact the job opportunities of healthcare professionals or dentists. In the present study, 32% of individuals did not feel any concern regarding job security, while 35% were somewhat concerned, and 24% reported being very concerned. A study by Singh et al. showed that 52.08% of respondents felt that AI could never replace dentists [17]. Studies by Kalaimani et al. [18], Yüzbaşıoğlu [19], and Swed et al. [20] indicated that respondents did not view AI as a threat to job opportunities for dentists.

Any technological advancement that occurs has a single aim: to enhance the current status of the human lifestyle by aiding it. In the present study, 61% of respondents reported that AI can somewhat enhance the diagnosis of dental ailments, while 31% strongly felt that it can help in both the diagnosis and treatment of dental diseases. Literature reports by Anil et al. have claimed that AI has the potential to facilitate easy, early, and precise diagnosis of dental caries [21].

It is imperative to highlight that disease management is based on a holistic approach that involves detailed clinical, radiological, and pathological examination. Simultaneously, it is essential to note that the final diagnosis, treatment plan, and prognosis often depend on radiological and pathological examination, whereas disease management is frequently clinical [22]. In the present survey, 71% of participants believed that AI can often enhance radiological diagnosis, while 64% believed AI can enhance pathological diagnosis. Similar findings were reported in a study by Singh et al., where AI was described as a "definitive diagnostic, prognostic, and treatment planning tool" by 62.3% of participants. They noted that AI is utilized in the radiographic diagnosis of dental caries, the diagnosis of soft tissue lesions, 3D implant positioning, forensic odontology, and even the diagnosis of oral pathological lesions [17].

Just like any other technology, AI has certain drawbacks. Privacy and data security are major concerns. AI algorithms are notorious for needing large amounts of patient data to function effectively, which exposes sensitive patient data to potential hacking and breaches by cybercriminals. Misdiagnosis or errors are also potential limitations [23]. A common comment in the open-ended questions of the present survey was that years of human experience cannot be replaced by machines, even with extensive programming. Therefore, AI can never fully replace healthcare professionals but can assist them.

AI demands large and high-quality datasets, which are often limited in the dentistry profession. Privacy regulations may restrict access to confidential patient information. Physicians struggle to understand and trust deep learning AI models due to their lack of transparency. If AI is trained on limited datasets, it may not achieve the required high accuracy, resulting in biases and inaccurate diagnoses. The inherent biases of AI algorithms, as well as liability issues, create ethical and legal challenges. Additionally, integrating AI into existing systems can be difficult, and some experts may be hesitant to adopt it due to unfamiliarity or fear of losing their employment.

## Conclusions

From the study, it is evident that AI is a promising tool that can aid and enhance the current state of dental sciences. It has the potential to improve compliance among both patients and dentists. AI could drastically alter dentistry by enhancing diagnostic accuracy, accelerating treatment planning, and improving patient outcomes. Advancements in AI may lead to more personalized care, better case management, and more efficient workflows in dental clinics. Deep learning and advanced imaging systems are examples of AI technologies that, when combined, have the potential to enhance treatment planning, increase patient engagement, and detect illnesses early. AI technologies are expected to improve, becoming more reliable, accessible, and widely used across various dental disciplines. In summary, AI presents exciting new opportunities for the field of dentistry. To fully capitalize on these benefits and ensure effective clinical practice, it is important to overcome limitations through careful adoption, ongoing research, and balanced integration.
